# Gastric Submucosal Fat Accumulation Is Associated with Insulin Resistance in Patients with Obesity

**DOI:** 10.1007/s11695-023-07014-2

**Published:** 2024-01-08

**Authors:** Tao Lu, Jianxun Kan, Xue He, Jialai Zou, Dandan Sheng, Yating Xue, Yan Wang, Lijian Xu

**Affiliations:** 1 Department of General Surgery, The Second Affiliated Hospital of Nanjing Medical University, 121 Jiang Jia Yuan Road, Nanjing, 210011 Jiangsu Province China; 2 Department of Pathology, The Second Affiliated Hospital of Nanjing Medical University, 121 Jiang Jia Yuan Road, Nanjing, 210011 Jiangsu Province China; 3 Department of Nuclear Medicine, The Second Affiliated Hospital of Nanjing Medical University, 121 Jiang Jia Yuan Road, Nanjing, 210011 Jiangsu Province China

**Keywords:** Fat accumulation, Gastric submucosal tissue, Obesity, Insulin resistance

## Abstract

**Purpose:**

Ectopic fat accumulation plays a significant role in obesity-related metabolic dysfunction, and few studies have reported an association between ectopic gastric fat and metabolic risk factors. We aim to fulfill this need by assessing the degree of gastric submucosal fat accumulation in pathologic sections of 190 sleeve gastrectomy specimens.

**Methods:**

Study patients were divided into two groups (D1 and D2) based on whether fat accumulation exceeded 1/3 of the submucosa of the stomach. Demographic and metabolic risk factors were compared between the two groups. Metabolic risk variables that might be associated with the degree of fat accumulation were screened in the original cohort. After balancing for possible confounders, the robustness of the correlations was assessed using binary and conditional logistic regression analyses.

**Results:**

All study patients had fat accumulation in the submucosa of the stomach. C-reactive protein (CRP), body mass index (BMI), visceral fat area (VFA), and insulin resistance (IR) were higher in the D2 group than in the D1 group in the original cohort (*P* < 0.05). Logistic regression analysis showed that BMI and IR may be associated with increased fat accumulation. After balancing variables other than obesity indicators and IR using propensity score matching, BMI and IR remained significantly different between the two groups (*P* < 0.05). Further analysis of the matched cohort using two logistic regression analyses showed that IR was an independent risk factor for increased fat accumulation.

**Conclusion:**

This study indicated that gastric submucosal fat accumulation was prevalent in patients with obesity and was associated with IR.

**Graphical Abstract:**

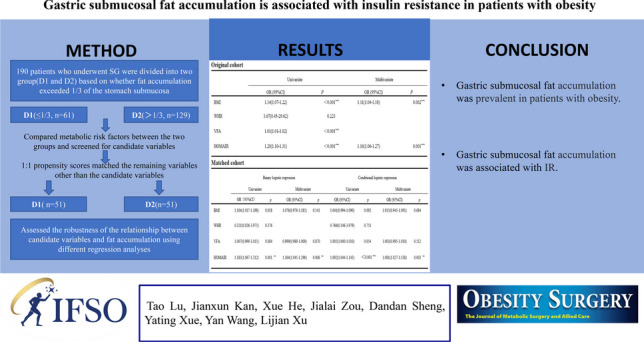

## Introduction

The prevalence of overweight and obesity has been increasing globally since 1980 [1]. By 2030, 51% of the population is expected to be obese [2]. Obesity is linked to several chronic illnesses, such as stroke, diabetes, coronary artery disease, hypertension, respiratory diseases, osteoarthritis, and gallstones [3–5]. As a result, individuals with obesity have a higher risk of early death and overall mortality rates [6, 7].

Obesity occurs when the body absorbs more calories than it consumes, leading to an abnormal accumulation or distribution of body fat. When the expansion capacity of subcutaneous fat is relatively insufficient to adapt to positive energy balance, fat will deposit in viscera and other ectopic organs [8–11], such as the liver, pancreas, heart, kidney, and colorectum [12–16]. Increased adiposity in ectopic organs is associated with cardiac and metabolic dysfunction [8, 17]. Therefore, assessing the fat content of different organs has important physiologic and clinical implications.

Fat accumulation in the stomach is considered ectopic and has been described as lipomatosis or lipohyperplasia of the stomach [18, 19]. We speculated that ectopic gastric fat is associated with metabolic risk factors (obesity, hypertension, diabetes, dyslipidemia, insulin resistance). However, there are very few studies on this topic. By assessing the extent of gastric submucosal fat accumulation in pathologic sections of sleeve gastrectomy specimens, we investigated the relationship between fat accumulation and metabolic risk factors to provide some information for studies on the specific role of ectopic fat deposition in the stomach.

## Materials and Methods

### Study Patients

A total of 190 patients with obesity (median age 32 years, range 15–70 years) who underwent sleeve gastrectomy from August 2020 to September 2022 were included. Exclusion criteria involved incomplete clinical data, serious infectious or immunological diseases, significant gastrointestinal disease, and poorly fixed pathological tissue sections.

### Anthropometric and Biochemical Measurements

Standard anthropometric methods were used to measure height and weight after removing shoes and coats. The formula for calculating BMI is as follows: weight (kg) / height^2^ (m^2^). After patients had fasted overnight, venous blood samples were taken and sent immediately to the hematology department for total cholesterol (TC), triglycerides (TG), high-density lipoprotein (HDL), low-density lipoprotein (LDL), fasting blood glucose (FPG), fasting insulin level (FINS), white blood cell (WBC), and C-reactive protein (CRP) level determination. The formula for calculating homeostasis model assessment of insulin resistance (HOMA-IR) was as follows: fasting blood glucose (mmol/L) × fasting insulin (mU/L) / 22.5 [20].

### Measurement of Visceral Fat Area

Dual-energy X-ray absorptiometry (DEXA) is a significant advancement in body fat assessment [21]. It is particularly useful in cases of severe malnutrition and overweight/obesity, as it provides accurate and reliable data on bone and soft tissue composition, including fat mass and lean body mass [22]. In the present study, we used DEXA to measure VFA. Hologic Discovery Wi scanner (S/N 86663) was used to perform the scans.

### Evaluation of Fat Accumulation in the Gastric Submucosa

**Fig. 1:**
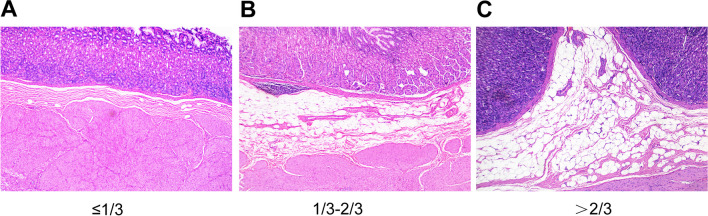
Three representative hematoxylin and eosin-stained sections of the gastric wall (× 4). The graphs showed the changes in gastric submucosal adipocyte accumulation in the samples studied: (A) adipocytes ≤ 1/3 of the submucosa; (B) adipocytes are 1/3–2/3 of the submucosa; (C) adipocytes > 2/3 of the submucosa

Archived gastric sections stained with hematoxylin and eosin (H&E) were evaluated blindly under a microscope. Compared with the proximal stomach, the distal antrum is thicker and submucosal fat is more common [23, 24], so we selected tissue sections from the antrum for observation. Since there was no grading method for the degree of submucosal fat accumulation in the stomach, we referred to the grading method for hepatic steatosis (0; ≤ 33%; 33–66%; > 66%) [25]. To make it easier to distinguish, we used 1/3 and 2/3 as the cutoffs instead of 33% and 66%. Two pathologists independently assessed the sections. If the assessments of the two pathologists were not in agreement, a third pathologist reviewed the slides and accepted the result that was in accord between the two of them (Fig. 1).

In our study, the degree of gastric fat accumulation was over 0 in all cases, and only one was more than 2/3, so we categorized the patients into a “D1” group (≤ 1/3) and a “D2” group (> 1/3) according to whether the fat accumulation exceeded 1/3 of the gastric submucosa.

### Statistical Analysis

Quantitative data were presented as means ± standard deviations (SD) for normal distributions and median (interquartile range) for non-normally distributed variables. Qualitative data were presented as frequencies. Continuous variables were compared using the *t*-test or Mann–Whitney *U* test, and categorical variables were compared employing the chi-square test.

Multifactor logistic regression forward stepwise was used to screen out candidate variables associated with degree of fat accumulation in the original cohort. Propensity score matching was used to balance possible confounders. Binary logistic regression and conditional logistic regression were used to analyze the matched data.

SPSS for Windows version 27.0 (SPSS, Chicago, IL, USA) was utilized to conduct the statistical analysis, and a *P*-value < 0.05 was considered statistically significant.

## Results

### Demographic and Metabolic Characteristics of Original Cohort

Before propensity score matching, 190 patients who underwent sleeve gastrectomy were included in the study. These patients were categorized into a “D1”group (≤ 1/3) and a “D2” group (> 1/3) according to whether the degree of submucosal fat accumulation in the stomach exceeded 1/3. Age, WBC, TC, TG, HDL, LDL, waist-to-hip ratio (WHR), history of diabetes mellitus, and history of hypertension did not show statistically significant differences between the two groups (*P* > 0.05). However, the sex ratio was significantly different (*P* < 0.05), and individuals in D2 group had higher values for CRP, BMI, VFA, and HOMA-IR compared to those in D1 group (*P* < 0.05) (Table 1).

**Table 1:** Demographic and metabolic characteristics of the original cohort

	Overall	D1 (≤ 1/3)	D2 (> 1/3)	*P*
*N*	190	61	129	
Gender (man/woman)	62:128	11:50	51:78	0.003^3^
Age (years)	32.57 ± 8.07	33.84 ± 9.03	31.97 ± 7.53	0.137^2^
C-reactive protein (mg/L)	1.40 (0.49–4.34)	0.63 (0.49–3.31)	1.67 (0.50–4.38)	0.036^1^
White blood cell (× 10^9^/L)	7.53 (6.48–8.95)	7.40 (6.41–8.98)	7.86 (6.54–8.92)	0.752^1^
Total cholesterol (mmol/L)	4.84 (4.08–5.35)	4.85 (4.14–5.37)	4.83 (4.07–5.34)	0.633^1^
Triglycerides (mmol/L)	1.72 (1.24–2.62)	1.62 (1.18–2.23)	1.88 (1.27–2.75)	0.122^1^
High-density lipoprotein (mmol/L)	1.03 (0.87–1.18)	1.07 (0.90–1.21)	1.02 (0.85–1.17)	0.156^1^
Low-density lipoprotein (mmol/L)	3.17 (2.46–3.69)	3.27 (2.51–3.65)	3.10 (2.44–3.74)	0.719^1^
History of hypertension (*n* (%))	87 (45.8)	30 (34.5)	57 (65.5)	0.519^3^
History of diabetes (*n* (%))	113 (59.5)	31 (27.4)	82 (72.6)	0.095^3^
BMI (kg/m^2^)	37.11 (33.11–41.01)	33.60 (31.24–37.22)	38.16 (34.46–44.04)	< 0.001^1^
Waist-to-hip ratio	1.21 ± 0.15	1.19 ± 0.16	1.22 ± 0.14	0.223^2^
Visceral fat area (cm^2^)	225.43 ± 59.15	202.23 ± 57.07	236.40 ± 57.10	< 0.001^2^
HOMA-IR	5.57 (2.84–9.11)	4.17 (1.58–6.07)	6.53 (3.54–10.93)	< 0.001^1^

Note: Data were expressed as mean ± standard deviation or median (interquartile range) for continuous variables and frequency (percentage) for categorical variables

Abbreviations: *BMI* body mass index, *HOMA-IR* homeostasis model assessment of insulin resistance

^1^Mann–Whitney test

^2^Student’s *t*-test

^3^Chi-square test

### Univariate and Multivariate Binary Logistic Regression Analyses of Original Cohort

We investigated the relationship between different variables and gastric submucosal fat accumulation using univariate logistic regression analysis, and all variables were subjected to further analysis using multifactorial logistic regression forward stepwise method. The results suggested that BMI and IR might be independent risk factors for fat accumulation in the gastric submucosa (Table 2).

**Table 2:** Univariate and multivariate binary logistic regression analyses of original cohort for association between gastric submucosal fat accumulation and different factors

	Univariate			Multivariate		
	*β*	OR (95%CI)	*P*	*β*	OR (95%CI)	*P*
Gender						
Man	Ref					
Woman	− 1.09	0.34 (0.16–0.71)	0.004**			
Age	− 0.03	0.97 (0.94–1.01)	0.140			
C-reactive protein	0.06	1.06 (0.98–1.15)	0.166			
White blood cell	0.00	1 (0.85–1.18)	1.000			
Total cholesterol	− 0.05	0.95 (0.71–1.27)	0.741			
Triglycerides	0.18	1.20 (0.91–1.57)	0.204			
High-density lipoprotein	− 1.19	0.31 (0.09–1.05)	0.060			
Low-density lipoprotein	− 0.03	0.97 (0.70–1.35)	0.847			
History of hypertension						
No	Ref					
Yes	− 0.20	0.82 (0.44–1.51)	0.519			
History of diabetes						
No	Ref					
Yes	0.52	1.69 (0.91–3.13)	0.096			
BMI	0.13	1.14 (1.07–1.22)	< 0.001***	0.10	1.11 (1.04–1.18)	0.002***
Waist-to-hip ratio	1.30	3.67 (0.45–29.62)	0.223			
Visceral fat area	0.01	1.01 (1.01–1.02)	< 0.001***			
HOMA-IR	0.18	1.20 (1.10–1.31)	< 0.001***	0.15	1.16 (1.06–1.27)	0.001***

Note: All variables in the univariate analysis were further analyzed using multivariate logistic regression forward stepwise method

Abbreviations: *BMI* body mass index, *HOMA-IR* homeostasis model assessment of insulin resistance

^*^P < 0.05; **P < 0.01; ***P < 0.001

### Demographic and Metabolic Characteristics of Matched Cohort

To minimize confounders, a 1:1 propensity score matching analysis was performed with gender, age, CRP, WBC, TC, TG, HDL, LDL, history of hypertension, and history of diabetes as covariates, with a caliper value of 0.01 without replacement.

The analysis included 51 patients from each group (D1 and D2) after propensity score matching. Gender, age, CRP, WBC, lipid levels, history of hypertension, and history of diabetes were not significantly different (*P* > 0.05) between the two groups.

Compared to pre-matching, VFA no longer showed a statistically significant difference (*P* > 0.05), but BMI and IR remained significantly higher in the D2 group than in the D1 group (*P* < 0.05). These results further suggested that BMI and IR might be linked to gastric submucosal fat (Table 3).

**Table 3:** Demographic and metabolic characteristics of the matched cohort

	Overall	D1 (≤ 1/3)	D2 (> 1/3)	*P*
*N*	102	51	51	
Gender (man/woman)	17:85	10:41	7:44	0.425^3^
Age (years)	32.15 ± 7.72	32.88 ± 7.96	31.41 ± 7.47	0.338^2^
C-reactive protein (mg/L)	1.31 (0.50–4.41)	0.99 (0.50–4.38)	1.99 (0.50–4.55)	0.396^1^
White blood cell (× 10^9^/L)	7.39 (6.37–8.97)	7.33 (6.37–8.99)	7.41 (6.44–8.96)	0.981^1^
Total cholesterol (mmol/L)	4.89 (4.21–5.45)	4.83 (4.15–5.36)	4.92 (4.23–5.73)	0.690^1^
Triglycerides (mmol/L)	1.62 (1.18–2.67)	1.62 (1.18–2.34)	1.58 (1.16–2.85)	0.888^1^
High-density lipoprotein (mmol/L)	1.04 (0.90–1.20)	1.03 (0.90–1.18)	1.05 (0.91–1.25)	0.738^1^
Low-density lipoprotein (mmol/L)	3.31 (2.46–3.69)	3.27 (2.56–3.69)	3.31 (2.42–3.77)	0.984^1^
History of hypertension (*n* (%))	39 (38.2)	22 (56.4)	17 (43.6)	0.308^3^
History of diabetes (*n* (%))	57 (55.9)	30 (52.6)	27 (47.4)	0.550^3^
BMI (kg/m^2^)	35.32 (32.73–39.54)	34.42 (32.00–37.50)	37.3 (34.30–40.40)	0.004^1^
Waist-to-hip ratio	1.19 ± 0.16	1.21 ± 0.17	1.18 ± 0.15	0.379^2^
Visceral fat area (cm^2^)	215.99 ± 52.64	206.90 ± 58.11	225.08 ± 45.30	0.081^2^
HOMA-IR	5.45 (2.78–8.80)	4.62 (2.06–6.65)	7.26 (3.68–11.42)	< 0.001^1^

Note: Data were expressed as mean ± standard deviation or median (interquartile range) for continuous variables and frequency (percentage) for categorical variables

Abbreviations: *BMI* body mass index, *HOMA-IR* homeostasis model assessment of insulin resistance

^1^Mann–Whitney test

^2^Student’s *t*-test

^3^Chi-square test

### Univariate and Multivariate Logistic Regression Analysis of Matched Cohort

To obtain robust results, we used two logistic regression models to analyze the matched data and included variables with *P* < 0.1 in the univariate analysis into the multivariate regression model. BMI and VFA were adjusted in the binary logistic regression model, and TG, BMI, and VFA were adjusted in the conditional logistic regression model. The results of both models indicated that IR was an independent risk factor for increased fat accumulation in the gastric submucosa (*P* < 0.05) (Table 4).

**Table 4:** Univariate and multivariate logistic regression analyses of matched cohort

	Binary logistic regression						Conditional logistic regression					
	Univariate			Multivariate			Univariate			Multivariate		
	*β*	OR (95%CI)	*P*	*β*	OR (95%CI)	*P*	*β*	OR (95%CI)	*P*	*β*	OR (95%CI)	*P*
Gender												
Man	Ref						Ref					
Woman	0.427	1.533 (0.534–4.405)	0.427				0.210	1.234 (0.554–2.747)	0.607			
Age	− 0.025	0.975 (0.926–1.026)	0.336				0.001	1.001 (0.965–1.040)	0.940			
C-reactive protein	0.025	1.025 (0.927–1.133)	0.631				0.014	1.014 (0.942–1.092)	0.716			
White blood cell	− 0.045	0.956 (0.785–1.165)	0.655				0.001	1.001 (0.860–1.166)	0.986			
Total cholesterol	0.107	1.113 (0.785–1.578)	0.548				0.007	1.007 (0.810–1.254)	0.947			
Triglycerides	0.060	1.062 (0.780–1.447)	0.701				0.223	1.250 (0.988–1.582)	0.063	0.043	1.044 (0.795–1.371)	0.756
High-density lipoprotein	− 0.084	0.919 (0.189–4.462)	0.917				− 0.690	0.502 (0.180–1.401)	0.188			
Low-density lipoprotein	0.050	1.051 (0.699–1.580)	0.810				− 0.043	0.958 (0.745–1.233)	0.739			
History of hypertension												
No	Ref						Ref					
Yes	− 0.417	0.659 (0.295–1.472)	0.309				− 0.098	0.906 (0.504–1.629)	0.742			
History of diabetes												
No	Ref						Ref					
Yes	− 0.239	0.788 (0.360–1.723)	0.550				0.222	1.249 (0.715–2.184)	0.435			
BMI	0.099	1.104 (1.017–1.198)	**0.018**	0.073	1.076 (0.976–1.185)	0.141	0.040	1.041 (0.994–1.090)	0.092	0.015	1.015 (0.945–1.091)	0.684
Waist-to-hip ratio	− 1.138	0.321 (0.026–3.975)	0.376				− 0.266	0.766 (0.148–3.979)	0.751			
Visceral fat area	0.007	1.007 (0.999–1.015)	0.084	− 0.001	0.999 (0.989–1.009)	0.870	0.005	1.005 (1.000–1.010)	0.054	0.002	1.003 (0.995–1.010)	0.512
HOMA-IR	0.168	1.183 (1.067–1.312)	**0.001**	0.152	1.164 (1.045–1.296)	**0.006**	0.088	1.092 (1.044–1.143)	** < 0.001**	0.078	1.081 (1.027–1.138)	**0.003**

Note: The variables with *P* < 0.1 in the univariate analysis were included into the multifactor logistic regression model

Abbreviations: *BMI* body mass index, *HOMA-IR* homeostasis model assessment of insulin resistance

Values in "**Bold**" indicate *P* < 0.05

## Discussion

In conclusion, we found that ectopic fat accumulation in the gastric submucosa was prevalent in patients with obesity. We screened metabolic risk factors related to gastric submucosal fat and found that BMI and IR may be potential related factors. After strict propensity score matching, IR showed a robust correlation with gastric submucosal fat. Although there have been many studies of ectopic fat accumulation in different organs, there are few studies involving ectopic fat accumulation in the stomach. As far as we know, this is the first study to investigate the relationship between gastric submucosal fat and metabolic risks based on pathological histology.

The gastric submucosa has a low amount of adipose tissue under normal conditions. It primarily consists of loose connective tissue with various types of cells, such as fibroblasts, macrophages, mast cells, and adipocytes, as well as blood vessels, lymphatic vessels, and nerve plexuses. Interestingly, pathology reports from bariatric metabolic surgery patients often mention increased submucosal fat in the stomach, leading us to speculate that gastric submucosal fat accumulation may be related to BMI or other obesity indicators. A previous researcher has assessed the degree of gastric wall fat infiltration using CT scans in 120 patients with renal colic and found that it is more common in individuals with a BMI greater than 25 kg/m^2^ [24]. Another study using similar methods found that gastric wall fat infiltration is more prevalent in overweight individuals, particularly those with increased visceral fat area and percentage of visceral fat [26]. Our study found similar results that fat accumulation in the submucosa of the stomach was prevalent in patients with obesity. Recently, Chumbalkar et al. [27] found that gastric submucosal fat accumulation was associated with BMI by comparing pathologic sections of 128 sleeve gastrectomy specimens with those of 89 Whipple procedure specimens. Although our study of the original cohort found that BMI may be associated with gastric fat accumulation, this correlation was not significant in the multifactorial regression model after rigorous propensity score matching. In the future, we will include more study patients and even people without obesity for in-depth analysis.

Adipose tissue plays a central role in metabolic dysregulation among patients with obesity. Adipocytes function as dynamic endocrine organs and nutritional sensors, strictly regulating the supply of energy. Excessive nutrient supply surpassing adipose tissue’s compensatory capacity leads to adipocyte hypertrophy and the initiation of pathological interactions between adipocytes and macrophages. As a result, adipocytes develop IR and chronically release free fatty acids, which have toxic effects on distant tissues such as the muscle, liver, pancreas, heart, and vascular beds [28]. Recent genetic and biochemical studies have revealed that dysregulation of metabolic mediators released by adipose tissue can lead to IR in other tissues [29]. Therefore, some scholars support the idea that IR is primarily a result of excess body fat [30–32]. Although the correlation between ectopic fat and IR has been revealed by an increasing number of studies [33–41], there is currently no research on the relationship between fat in the stomach and IR. In the present study, the results of the original and matched cohort showed that gastric submucosal fat accumulation was associated with IR. This finding suggested that the effects of increased gastric submucosal fat on the body may be negative. If increased fat accumulation is found in gastric pathology sections, it should be given attention and described. The specific role of ectopic fat accumulation in the stomach requires further research to elucidate. Our study revealed a potential relationship between gastric submucosal fat and IR, but biological systems are complex and there may be other variables associated with gastric submucosal fat, so further studies are needed to identify and understand these factors. In addition, ectopic fat in different organs is associated with IR, and gastric fat is only a part of ectopic fat pools, so gastric fat can only explain part of IR. To fully understand the relationship between ectopic fat and IR, further investigations are needed to determine how much each ectopic fat organ contributes to IR.

This study has some limitations that should be taken into account. Firstly, based on the cross-sectional design, we can only estimate correlation, not causation. Secondly, the sample size of the study is limited, and we will strive to include more cases in the future and conduct multicenter validation to enhance the generalizability of the findings. Thirdly, the assessment of tissue sections through microscopy is subjective, but efforts were made to minimize potential errors by employing blinded methods during the evaluation process.

## Conclusion

Gastric ectopic fat was prevalent in the submucosal layer of patients with obesity, similar to fat accumulation in the liver, and associated with IR. Our observations provide some basis for future studies on the relationship between gastric ectopic fat and metabolic diseases. However, the exact mechanism of gastric ectopic fat deposition and its impact on health remain to be fully elucidated.

## Data Availability

The data that support the findings of this study are available upon request.

## Appendix

The general procedures for collection and processing of pathologic sections of the sleeve stomach were as follows:

1. Specimen fixation: separated sleeve gastrectomy specimens were immediately fixed by formalin immersion.
2. Tissue collection: four pieces of tissue (cutting edge, fundus, antrum and body) were taken from each sleeve gastrectomy specimen
3. Tissue fixation: continue to fix the collected tissue with formalin immersion.
4. Tissue dehydration: the collected tissues were dehydrated to remove the fixative.
5. Tissue embedding: the processed tissue specimens were embedded in a wax block to form a tissue block.
6. Section preparation: The tissue block was cut into very thin sections (usually 4–6 microns) using a microtome, and the sections were then placed on slides.
7. Staining: Hematoxylin and eosin (HE) staining was used to highlight tissue structure and cellular features.
8. Coverslip encapsulation: The stained slides were covered with coverslips and encapsulated with clear adhesive to protect the sections.
